# Development of a scale to assess cancer stigma in the non-patient population

**DOI:** 10.1186/1471-2407-14-285

**Published:** 2014-04-23

**Authors:** Laura AV Marlow, Jane Wardle

**Affiliations:** 1 Cancer Research UK Health Behaviour Research Centre, Department of Epidemiology and Public Health, UCL, Gower Street, London WC1E 6BT, UK

**Keywords:** CASS, Cancer, Scale, Measure, Blame, Attitudes

## Abstract

**Background:**

Illness-related stigma has attracted considerable research interest, but few studies have specifically examined stigmatisation of cancer in the non-patient population. The present study developed and validated a Cancer Stigma Scale (CASS) for use in the general population.

**Methods:**

An item pool was developed on the basis of previous research into illness-related stigma in the general population and patients with cancer. Two studies were carried out. The first study used Exploratory factor analysis to explore the structure of items in a sample of 462 postgraduate students recruited through a London university. The second study used Confirmatory factor analysis to confirm the structure among 238 adults recruited through an online market research panel. Internal reliability, test-retest reliability and construct validity were also assessed.

**Results:**

Exploratory factor analysis suggested six subscales, representing: Awkwardness, Severity, Avoidance, Policy Opposition, Personal Responsibility and Financial Discrimination. Confirmatory factor analysis confirmed this structure with a 25-item scale. All subscales showed adequate to good internal and test-retest reliability in both samples. Construct validity was also good, with mean scores for each subscale varying in the expected directions by age, gender, experience of cancer, awareness of lifestyle risk factors for cancer, and social desirability. Means for the subscales were consistent across the two samples.

**Conclusions:**

These findings highlight the complexity of cancer stigma and provide the Cancer Stigma Scale (CASS) which can be used to compare populations, types of cancer and evaluate the effects of interventions designed to reduce cancer stigma in non-patient populations.

## Background

Stigma is an attribute that discredits a person, reducing them “from a whole and usual person to a tainted, discounted one” (p.12) [1]. This highlights two important components of stigma: the characteristic that makes a person ‘different’ and the devaluation of the person on the basis of this difference [2]. Health-related stigma refers to stigmatisation of an illness, which can be applied to an individual or a group of people with the illness, as well as to the illness more generally [3]. It is “characterized by exclusion, rejection, blame or devaluation that results from experience, perception or reasonable anticipation of an adverse social judgment about a person or group” (p.280) [4]. There is widespread agreement that illness stigmatisation is not stable, but influenced by social attitudes that differ across cultures and change over time [5].

Most of the literature exploring health-related stigma has focused on a small group of illnesses: leprosy, epilepsy, HIV/AIDS and mental illness [6]. Cancer has attracted less research attention, although it is often described as a stigmatised condition (http://livestrongblog.org/2010/02/01/cancer-stigma), and perceptions of stigma have been identified as a concern among cancer patients. Patients sometimes feel avoided by others once they have received a cancer diagnosis [7-9] and fear of stigmatisation can be a barrier to disclosure of a cancer diagnosis [8,10]. Work exploring stigma among cancer patients has focused on lung cancer, with patients who are smokers feeling blamed because of the perception that their illness is self-inflicted [11].

Few studies have explored stigma towards cancer in the non-patient population, although there are several reasons why this is important. The availability of cancer detection and prevention procedures (e.g. screening and HPV vaccination) means that people need to consider the possibility of a cancer diagnosis in the context of making preventive health decisions and fear of stigmatisation has been identified as a potential barrier to self-examination, screening, and delayed presentation of cancer symptoms [12-18]. It is also possible that the growing number of public health campaigns designed to educate the public about behavioural risk factors for cancer (e.g. smoking, obesity, Human Papillomavirus) could generate stigma by implying that cancers are avoidable [11].

The aim of the present study was to develop a Cancer Stigma Scale (CASS) for use in non-patient populations. Being able to measure the stigma of cancer would make it possible to identify the extent to which stigma exists, to monitor changes in perceptions of cancer as a result of public health campaigns or media attention and to help identify risk factors for more stigmatised beliefs.

### A multidimensional concept

Stigma is considered to be a multidimensional concept. Jones et al. identified six components of health-related stigma which apply to varying degrees depending on the illness of interest [19]. The first component, ‘peril’, relates to perceived danger from the stigmatised person, for example if their illness is considered contagious (e.g. HIV/AIDs) or they are considered dangerous (e.g. some mental illnesses). Interacting with those who are ill also raises awareness of personal mortality, resulting in anxiety and the need to see those who have the illness as different [20]. This side of peril is particularly relevant to cancer: ‘[The] dying cancer patient may make us starkly and disagreeably aware that a similar fate can befall us’ (p.66, [19]).

The second component, ‘course’, refers to changes in the illness over time, with conditions that are progressively crippling, chronic and incurable being more stigmatised. This is similar to the component of ‘stability’, identified by others [21]. If beliefs about the success of cancer treatments become more optimistic and the chances of long-term survivorship are seen as higher, the course-related element of stigma could be reduced.

The third component is ‘origin’. This relates to when and how the illness is believed to have come about. A particularly relevant aspect of this is the attributions of perceived responsibility, because when a person is believed to have caused their illness, the associated stigma is greater [19]. Again this is supported by other stigma theorists [5,21]. It may become increasingly relevant for cancer as the lifestyle determinants are more widely recognized. There is already evidence that lung cancer patients believe that the well-established link between smoking and their type of cancer contributes to stigmatisation [8], and more blame is attributed to patients with lung cancer than patients with leukaemia, breast, bowel or cervical cancer [22].

The three remaining components are ‘concealability’ (whether an illness can be hidden from others), ‘disruptiveness’ (whether it disrupts usual interactions), and ‘aesthetics’ (described as a primitive response by the perceiver to a non-concealable mark that makes the person less ‘pleasing on the eye’). Similar aspects are identified by other theorists, including Crocker et al. [5] who suggested the ‘visability’ of a stigma creates a schema through which all other aspects of the person are viewed. Some cancers do not have any visible signs, i.e. they are concealable. However, cancer treatment can result in more visible signs such as alopecia or a colostomy bag, and several studies have shown that these signs contribute to feelings of stigmatisation [23,24].

These six components help to highlight the aspects of an illness that may contribute to it being stigmatised. Each of the components could be considered from the perspective of the perceiver or the target [2]. Traditionally, studies considering behavioral aspects of stigma have assessed interpersonal avoidance and social distance [25-27], but attitudes towards discrimination (e.g. employment law, access to financial services) have also been considered [28,29].

### Measuring cancer-related stigma

In recent years there has been an increase in research into perceptions of stigma among cancer patients, but little systematic research into the general public’s attitudes. In a review of 38 articles exploring stigma and cancer, the majority focused on the cancer patient’s experience [30], with only seven studies in non-patient samples, and these were mostly qualitative investigations. To our knowledge, at the time of conducting this work no scales were available for assessing cancer stigma in the non-patient population. A 2006 review of illness-related stigma identified 24 scales, but none of them assessed cancer-related stigma [6]. Although these scales have traditionally been used to indicate stigma, stigma of cancer was expected to be more subtle than with other illnesses and many of the items traditionally used are unlikely to be appropriate because of the non-contagiousness of cancer (e.g. I would share a plate with someone with *cancer*). Twenty years ago a measure of cancer attitudes was developed that included some items related to stigma (the Cancer Attitudes Inventory [31]; available in [32]). However, it was designed to be unidimensional, and did not reflect the different aspects of stigma that might be relevant to cancer. More recently, the Cataldo Lung Cancer Stigma Scale (CLCSS) was developed for lung cancer patients [33]. Adapted from an HIV Stigma Scale, the CLCSS is a multidimensional measure with four subscales assessing stigma and shame, social isolation, discrimination and smoking. We used a similar approach to develop a multidimensional scale of cancer stigma, drawing on measures of stigma in other illnesses and on the literature exploring perceptions of stigma in cancer patients.

## Methods

### Development of an item pool

An item pool was developed on the basis of previous research into illness-related stigma in the general population and patients with cancer. Illness-related stigma scales were identified through a systematic review [6] that had used the search terms ‘stigma’ or ‘discrimination,’ and ‘scales’, ‘measurement’ or ‘assessment’. Overall, 24 different measures had been used to assess stigma in the general population, relating to leprosy, HIV/AIDS, mental illness, epilepsy and skin disease. For five of the studies, we could not access the scales, so items from 19 quantitative studies were included in the item pool. Studies exploring perceptions of cancer-related stigma were identified through a second systematic review [29] which used the search terms ‘stigma and cancer’, ‘stigma, psychosocial and cancer’ and ‘discrimination and cancer’. The review identified 38 studies of which three were not available, and 14 either used indirect measures of stigma (e.g. GP referral or internet use) or assessed a very specific area (e.g. attitudes to HPV or alopecia). Relevant items were adapted from the remaining 21 studies. Thirty-five items from the Cancer Attitudes Inventory (CAI) were also included in the initial item pool [31]. In total, 481 items were extracted from 41 studies that had used qualitative or quantitative methods to assess stigma in patient and non-patient samples (see Additional file 1).

Items were organized into themes and inspected to ensure that all relevant aspects of stigma (as identified by Jones et al. 1980) were covered. Duplicate items were deleted. The item pool was then discussed with a panel of cancer researchers (n = 7, post-doctoral fellows and senior researchers, with backgrounds in behavioural science and psychology). The quality of each item was discussed in a single meeting following which further changes were made to i) remove ambiguous items, ii) simplify wording, iii) remove ‘loaded’ items, e.g. ‘suffering’ with cancer and ‘cancer patient’, iv) remove personalized items e.g. ‘if I had cancer…’, and v) ensure that there were some positively worded items.

The refined item pool included 84 items. All attitude items were phrased so that a 6-point response scale of disagree strongly, disagree moderately, disagree slightly, agree slightly, agree moderately and agree strongly, would be appropriate. Response options for anticipated emotional and behavioral reactions to someone with cancer were definitely not, probably not, possibly not, yes possibly, yes probably and yes definitely. A ‘not sure’ option was also offered for all items; this was positioned to the right of the 6-point scale (rather than as a mid-point) and was separated by a vertical dotted line to minimize the chance of people using it because they wanted to avoid thinking about the question.

The first item set was given to an opportunistic sample (n = 57 students) to ensure that questions were answerable and wording was clear. This resulted in deletion of two items because a large proportion of participants (more than 20%) found them too difficult to answer. Small changes were also made to the wording of several items.^a^

### Testing the item pool

To explore the structure of the items and to test validity and reliability, data were collected from two samples: postgraduate students (study 1) and online panel participants (study 2). Both groups completed the questionnaire online and anonymously. We chose this modality because there is some suggestion that web-based data collection can reduce the social desirability pressures of responding to sensitive questions [34,35].

#### Study 1 – Student sample

##### Methods

A link to the questionnaire was sent via email to all postgraduate students at a University in London. Our target was to recruit a minimum of 300 students in order to have a ‘good’ sample size for running factor analysis [36]. The online survey was closed two weeks after the recruitment email was sent. Two to three weeks after original completion of the questionnaire students were sent a second email asking them to complete the survey again for test-retest reliability. The email included an ID number which they were asked to enter and this allowed us to match the two sets of results. The study was approved by the UCL Research Ethics Committee (ref: 0496/007). Students were offered entry into a prize draw to win £100. Entry into an additional prize draw to win £50 was offered for completing the survey a second time.

As well as completing the 82 stigma items, participants completed i) the Level of Familiarity Questionnaire, originally designed to assess familiarity with mental illness [37] but adapted here for cancer, ii) a 10-item measure of social desirability (The M-C 2(10), [38]), and iii) a question from the Cancer Awareness Measure [39], which asks participants to ‘put the following things in order of how much you think they contribute to cancer’ (lifestyle, chance, aging, environmental factors, and genetic inheritance). Based on findings from the stigma literature, we expected cancer-related stigma to be lower in people who were more familiar with the disease [37], and to be higher in those who attributed cancer more strongly to lifestyle [22]. Gender, age, and subject of study were also reported. Based on the literature exploring stigma of other illnesses, we expected stigma to vary by gender and age [40,41].

Data were analyzed in SPSS version 15.0. Exploratory factor analysis was used to examine the underlying factors in the questionnaire. On the basis of these results, scales assessing different aspects of cancer stigma were computed. Internal reliability, test-retest reliability and construct validity were assessed for each of the factors. Correlations between factors were also examined.

##### Results

Overall 473 postgraduate students completed the questionnaire, of whom three quarters (72%) were female, with a mean age of 29.1 (range 20–75). Current subject of study was coded according to the university faculties list and categorized into: arts and humanities (n = 144), engineering and mathematics (n = 140), life sciences and medicine (n = 181). A small proportion of respondents reported that they had had cancer (3%), had lived with someone who had cancer (7%), or had worked in a job which involved providing services to someone with cancer (12%). The overwhelming majority responded yes to the item ‘a friend of the family has had cancer’ or ‘I have a relative who has cancer’ (91%). Fifteen percent had never been around anyone with cancer. Lifestyle was rated as the main cause of cancer by 37% of participants. Social desirability was recoded into a binary variable for ease of presentation, with 53% scoring low (mean: 0–5) and 47% high on social desirability (mean: 6–10).

### Item distributions

Ten items were deleted because a large proportion of respondents (more than 20%) indicated that they were unsure (i.e. they could not agree or disagree with the item). According to Clark & Watson [42], it is important to inspect the distribution of individual items and delete items that have a highly skewed distribution. Item distributions were examined, and when fewer than 5% or more than 95% of respondents agreed, the item was excluded (a further six items). Where >20% of the remaining 66 items were missing, data from that respondent were excluded from further analysis (n = 9).

### Factor analysis

The 66 items were entered into a principal components analysis. Inspection of the scree plot suggested that the data were best represented by an 8-factor solution. This accounted for 46% of the variance in the 66 items, and the factors had eigenvalues of: 10.59, 6.36, 2.96, 2.76, 2.36, 1.98, 1.70 and 1.66. The Kaiser value was .86 and Bartlett’s test was significant, indicating that factor analysis was appropriate. An oblique rotation (Promax) was used, because the factors were expected to be correlated. The eight factors assessed: *Awkwardness* (e.g. I would find it difficult being around someone with cancer), *Severity* (e.g. Once you’ve had cancer you’re never ‘normal’ again), *Avoidance* (e.g. I would distance myself physically from someone with cancer), *Policy Opposition* (e.g. More government funding should be spent on the care and treatment of those with cancer, reversed), *Personal Responsibility* (e.g. A person with cancer is accountable for their condition), *Pity* (e.g. I would feel sorry for someone with cancer), *Financial Discrimination* (e.g. It is acceptable for banks to refuse to make loans to people with cancer) and *Fear* (e.g. Cancer is more frightening than most other diseases).

Items loading < .4 or loading equally onto multiple factors (with < .10 difference between the loadings) were rejected (22 items). This included all items that loaded on the *Fear* factor, all of which had low factor loadings (.33-.39), therefore *Fear* was not considered in further analyses. The item-total correlations were inspected and one item was deleted because the correlation was <0.3 [43]. At this stage we also took the decision to exclude the *Pity* factor. Items assessing pity were included in the initial item pool because previous studies on stigma had included this construct. However, 2 of the 4 items remaining in this subscale had borderline factor loadings (<.45) and the scale had a limited range. Most respondents scored very highly (94% scored >4, mean = 4.98, standard deviation = 0.74) and no-one scored below 2. We therefore decided that pity was not appropriately discriminating in the present scale.

Following these exclusions we were left with 39 items, of which between 3 and 12 loaded on each of the remaining six factors. To ensure the scale would not be too long and to control participant burden, we excluded additional items with the lowest factor loadings (for factors with multiple items). Content validity was also taken into account, so in one instance an item on the *Avoidance* subscale which was considered important (I would try to avoid a person with cancer, loading = .50) remained in place of one with a slightly higher loading (I feel threatened by someone with cancer, loading = .54).

**Figure 1:**
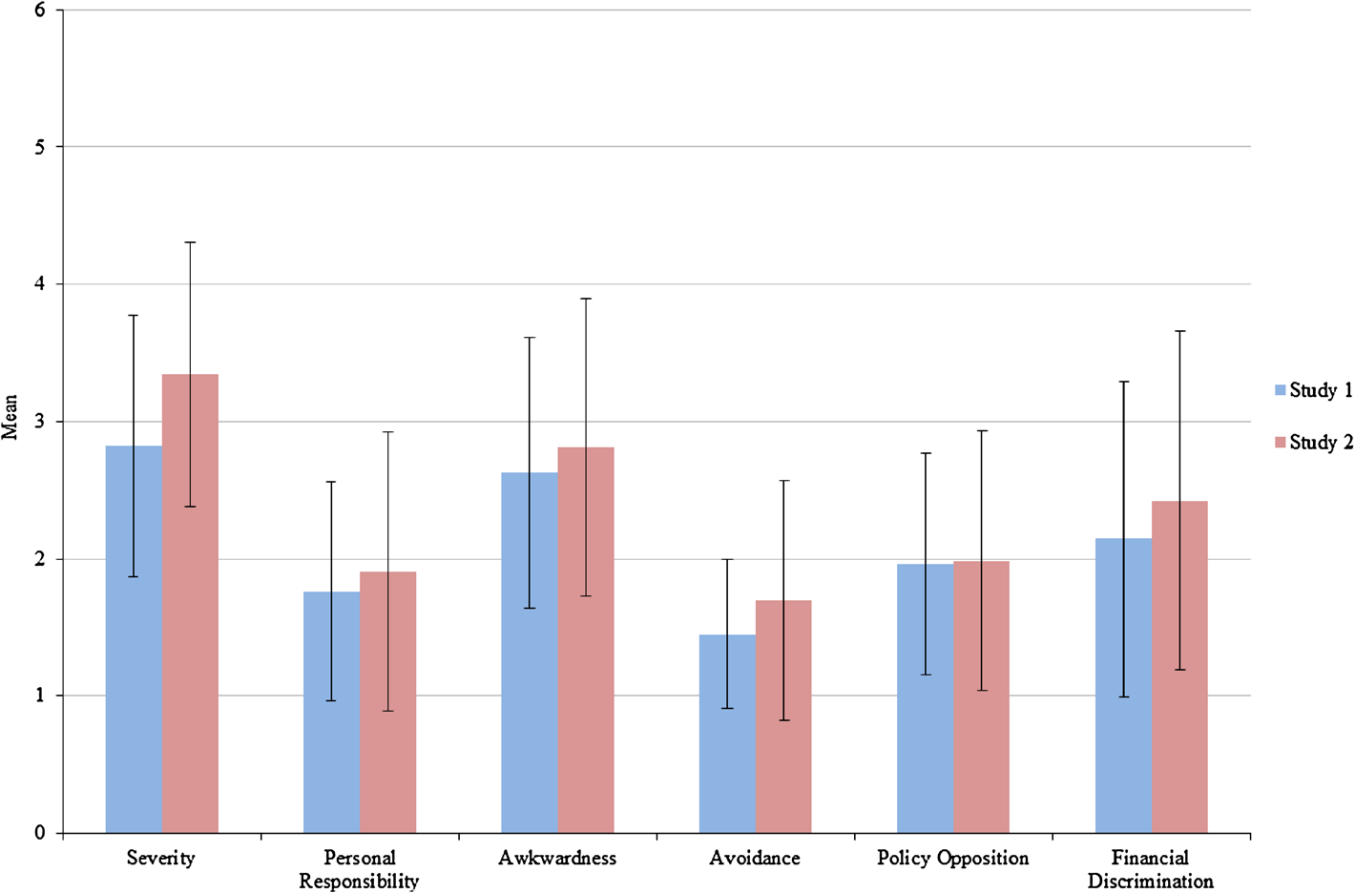
Mean scores and standard deviations for each factor across the two studies.

The final scale included 26 items, with 3 to 5 assessing: *Awkwardness*, *Severity*, A*voidance*, *Policy Opposition*, *Personal Responsibility* and *Financial Discrimination* (see Table 1). Scores were calculated by taking the mean of the final item list. These scores correlated highly with Bartlett’s factor scores, supporting our decision to shorten the scales (r = .93 to .98, p < .001). Figure 1 shows the means and standard deviations for each factor. Higher scores indicated more negative attitudes for all factors. There were significant inter-correlations between most factors (see Table 2), ranging from small to medium, r = .11 to r = .46. The exception was *Policy Opposition*, which was not correlated with *Severity* or *Awkwardness*.

**Table 1:** Exploratory factor analysis of stigma-related cancer

	**Factor loading**
**Awkwardness**	
I would feel at ease around someone with cancer (R)	-0.82
I would feel comfortable around someone with cancer (R)	-0.80
I would find it difficult being around someone with cancer	0.67
I would find it hard to talk to someone with cancer	0.67
I would feel embarrassed discussing cancer with someone who had it	0.62
**Severity**	
Once you’ve had cancer you’re never ‘normal’ again	0.78
Having cancer usually ruins a person’s Career	0.68
Getting cancer means having to mentally prepare oneself for death	0.65
Cancer usually ruins close personal relationships	0.62
Cancer devastates the lives of those it touches	0.60
**Avoidance**	
If a colleague had cancer I would try to avoid them	0.70
I would distance myself physically from someone with cancer	0.68
I would feel irritated by someone with cancer	0.66
I would feel angered by someone with cancer	0.55
I would try to avoid a person with cancer	0.50
**Policy opposition**	
More government funding should be spent on the care and treatment of those with cancer (R)	0.82
Increased spending on cancer services is a waste of money*	-0.71
The needs of people with cancer should be given top priority (R)	0.63
We have a responsibility to provide the best possible care for people with cancer (R)	0.60
**Personal responsibility**	
A person with cancer is liable for their condition	0.81
A person with cancer is accountable for their condition	0.78
If a person has cancer it’s probably their fault	0.73
A person with cancer is to blame for their condition	0.70
**Financial discrimination**	
It is acceptable for banks to refuse to make loans to people with cancer	0.89
Banks should be allowed to refuse mortgage applications for cancer-related reasons	0.77
It is acceptable for insurance companies to reconsider a policy if someone has cancer	0.63

R = Item was reversed.

*Item deleted following CFA.

**Table 2:** Correlations and reliability for each factor

	**Severity**	**Personal responsibility**	**Awkwardness**	**Avoidance**	**Policy opposition**	**Financial discrimination**
**Study 1**						
Correlations						
Personal responsibility	.11*	-	-	-	-	-
Awkwardness	.36**	.17**	-	-	-	-
Avoidance	.30**	.37**	.46**	-	-	-
Policy opposition	.00	.28**	.04	.21**	-	-
Financial discrimination	.15**	.27**	.15**	.23**	.35**	-
Internal reliability	.73	.87	.81	.77	.78	.82
Test-retest reliability	.82**	.72**	.80**	.77**	.76**	.72**
**Study 2**						
Correlations						
Personal responsibility	.24**	-	-	-	-	-
Awkwardness	.27**	.33**	-	-	-	-
Avoidance	.32**	.64**	.49**	-	-	-
Policy opposition	-.17**	.35**	.34**	.39**	-	-
Financial discrimination	.19**	.37**	.25**	.43**	.23**	-
Internal reliability	.76	.91	.81	.88	.78	.80

*p < .05, **p < .01.

### Internal reliability and test-retest reliability

Internal reliability was adequate for all the factors (Cronbach’s α = 0.73-0.87). The questionnaire was completed for a second time by 54% of the respondents (n = 249) and correlations between time 1 and time 2 scores were significant for all the factors (r = .72-.82, all p’s < .001).

### Construct validity

Differences in mean scores for each component by gender, age, subject being studied, belief that lifestyle is the main cause of cancer, experience of cancer, and social desirability were explored (reported in Table 3). Female students had lower means for *Personal Responsibility*, *Avoidance, Policy Opposition* and *Financial Discrimination*; indicating lower stigma than male students. Compared with younger students (aged 20–29 years), those over 30 years had lower *Personal Responsibility* and *Awkwardness* scores. Students studying for life science or medical qualifications had lower *Awkwardness* scores than those studying other subjects (e.g. arts, humanities, maths, or engineering). Students who believed that lifestyle was the main contributor for cancer reported higher *Personal Responsibility* than those who did not rank it as the main contributor. Because close experience with cancer was quite low in this group, we used the item relating to ever having been around a person with cancer as an indicator of some experience with the illness. Students who had been around someone with cancer had lower *Personal Responsibility* and *Awkwardness* scores than those who reported never having been around someone with cancer, and there was also a trend towards lower *Avoidance* in this group (bordering on significant, p = .057). High social desirability (scoring 6–10) was associated with lower scores for *Avoidance* and *Financial Discrimination*. Re-running the above analyses adjusting for social desirability produced very similar results, with no changes to which independent variables were significant.

**Table 3:** Construct validity in the student sample

	**Severity**	**Personal responsibility**	**Awkwardness**	**Avoidance**	**Policy opposition**	**Financial discrimination**
Sex						
Male	2.78	1.88	2.60	1.55	2.26	2.47
Female	2.84	1.71	2.64	1.41	1.85	2.01
t-test (p value)	-0.60 (.552)	2.02 (.044)	-0.38 (.704)	2.31 (.022)	4.51 (<.001)	3.58 (<.001)
Age						
20-29 years	2.82	1.82	2.72	1.47	1.98	2.20
30+ years	2.82	1.62	2.42	1.39	1.93	2.02
t-test (p value)	-0.01 (.992)	2.68 (.008)	3.10 (.002)	1.51 (.132)	0.58 (.561)	1.49 (.137)
Subject studied						
Life sciences/medical	2.74	1.74	2.46	1.40	1.95	1.11
Other	2.89	1.77	2.75	1.49	1.98	1.18
t-test (p value)	-1.55 (.121)	-0.41 (.683)	-3.08 (.002)	-1.82 (.069)	0.36 (.716)	-0.49 (.624)
Belief that lifestyle is cause						
No	2.83	1.65	2.62	1.45	2.00	2.12
Yes	2.81	1.95	2.64	1.45	1.90	2.17
t-test (p value)	0.22 (.826)	-3.76 (<.001)	-0.20 (.838)	-0.14 (.886)	1.40 (.163)	-0.40 (.692)
Ever been around a person with cancer						
Yes	2.81	1.71	2.59	1.43	1.94	2.13
No	2.88	2.00	2.85	1.56	2.08	2.23
t-test (p value)	-.58 (.563)	-2.81 (.005)	-2.15 (.032)	-1.91 (.057)	1.40 (.163)	-0.66 (.511)
Social desirability						
Low score (0–5)	2.83	1.79	2.66	1.50	2.01	2.26
High score (6–10)	2.81	1.73	2.59	1.40	1.91	2.01
t-test (p value)	0.22 (.827)	0.85 (.397)	0.83 (.409)	2.09 (.037)	1.43 (.154)	2.22 (.027)

#### Study 2 – online panel sample

##### Methods

The aim of study 2 was to confirm the factor structure, as well as the reliability and validity of the scales in a different sample. Participants were recruited through an online market research agency (S*urvey Sampling*) which holds a panel of over 200,000 UK residents who have consented to being contacted by email about online research studies. Panel members are offered points which can be exchanged for small rewards (e.g. airmiles). A random selection of participants were sent an email inviting them to take part in our study. Our target was to recruit at least 200 participants. This was set lower than in study 1 because there were fewer items by this stage. We set several quotas to ensure equal numbers of men and women, younger (<35) and older, and with or without a university degree. Participants completed the 26 stigma items, and also the Level of Familiarity (with cancer) questionnaire [37], and a measure of social desirability [38]. They reported gender, age, education and ethnicity. The study was approved by the UCL Research Ethics Committee (ref: 0496/007). Confirmatory Factor analysis was run in Mplus 7.11.

##### Results

Data were collected from 256 participants over 18 days. Half were male (51%) and the mean age was 38 (range 16–80 years). Just under half were single (45%), 44% were married or cohabiting, and the rest were separated/divorced or widowed (9% and 3% respectively). All participants had at least some educational qualifications: 27% indicated basic school-based exams (GCSEs) as their highest qualification, 30% had A-levels or a qualification below degree, and 29% had a university degree. The majority were from white ethnic backgrounds (80%). Very few had lived with someone who had cancer (9%), provided services to people with cancer (6%), or had experienced cancer themselves (4%). About three quarters (72%) reported that a close^b^ friend or family member had had cancer, and only 22% said they had never been around anyone with cancer. Cases where more than 20% of the 66 variables were missing were excluded from further analysis (n = 16).

### Confirmatory factor analysis

The 26 stigma items were entered into a six-factor confirmatory factor analysis (CFA) model. Indicators of each factor only loaded on their own factor. Measurement errors between indicators were assumed to be uncorrelated, but factors were allowed to correlate with each other. Z-scores for skewness and kurtosis were significant for some items (-10.73 to 11.65 and -2.95 to 11.94 respectively) so a robust maximum likelihood model (MLM) was used. We considered multiple measures of model fit using criteria [44]; CFI and TLI (>0.90), SRMR (>0.06) in combination with RMSEA (>0.05). In the first model, some of the parameters suggested a poor fit (CFI and TFI < .09, see Table 4), so the modification indices were closely inspected. On the basis of inspection, one item was removed because it loaded onto several factors (Increased spending on cancer services is a waste of money) and correlated residual errors were allowed between two items within the A*wkwardness* factor (I would feel at ease around someone with cancer and I would feel comfortable around someone with cancer). The close similarity in wording of these two items could explain the significance of their correlated residual errors. In the final model (χ^2^_SB_(259) = 379.624, p < .001), which included 25 items and 6-factors there was an improved fit; CFI = 0.942, TLI = 0.933, SRMR = 0.064, RMSEA = 0.052. Standardised factor loadings ranged from 0.45-0.90 and were all significant at p < .001.

**Table 4:** Results of confirmatory factor analyses testing the generalizability of a 6-factor model of cancer stigma (n = 169)

**Model**	**Items**	***χ***^**2**^_**SB**_	**df**	**CFI**	**TFI**	**SRMR**	**RMSEA**
1. Unconstrained	26	562.80***	284	0.86	0.86	0.087	0.076
2. 1 item excluded^1^	25	464.00***	260	0.90	0.89	0.071	0.068
3. 1 item excluded^1^ and correlated residuals allowed^2^	25	379.63***	259	0.94	0.93	0.064	0.052

^1^Item excluded from the Policy Opposition factor (Increased spending on cancer services is a waste of money) because the modification indices suggested it also loaded well onto several other factors.

^2^Correlated residuals were allowed between I would feel at ease around someone with cancer and I would feel comfortable around someone with cancer.

***p<.001.

### Means, correlations and internal reliability

Scores for each factor were calculated in the same way as study 1, and the means and standard deviations are shown in Figure 1 (NOTE: for study 2 the *Policy Opposition* Score was based on 3 rather than 4 items). The pattern of inter-correlations between factors was largely similar to study 1 (see Table 2), ranging from small to medium for most pairs (r = -.17-.49), but large for *Personal Responsibility* and *Avoidance* (r = .64). Internal reliability ranged from adequate to good (Cronbach’s α = 0.76-0.91).

### Construct validity

Differences in mean scores for each component by gender, age, belief that lifestyle is the main cause of cancer, experience of cancer and social desirability were explored (reported in Table 5). Women had lower means for *Avoidance* and *Policy Opposition*, there was also a tendency towards lower *Personal Responsibility* (bordering on significant, p = .059). Older age was associated with lower *Personal Responsibility*, *Awkwardness*, *Avoidance, Policy Opposition* and *Financial Discrimination*. Participants from ethnic groups other than white-British scored higher on *Personal Responsibility*, *Avoidance, Policy Opposition* and *Financial Discrimination*. Higher educational level was associated with higher *Policy Opposition* scores. Participants who reported that a friend or family member they were close to had had cancer scored lower on A*wkwardness*. High social desirability was associated with reporting lower *Awkwardness*, *Avoidance, Policy Opposition* and *Financial Discrimination.* We repeated the above analyses controlling for social desirability. Age was no longer associated with A*voidance* or *Financial Discrimination,* but all other findings remained significant.

**Table 5:** Construct validity in the non-student sample

	**Severity**	**Personal responsibility**	**Awkwardness**	**Avoidance**	**Policy opposition**	**Financial discrimination**
Sex						
Male	3.23	2.03	2.84	1.86	2.14	2.50
Female	3.45	1.78	2.78	1.52	1.83	2.34
t-test (p-value)	-1.77 (.077)	1.89 (.059)	0.47 (.643)	2.98 (.003)	2.59 (.010)	0.98 (.327)
Age						
16-34 years	3.42	2.17	3.18	1.83	2.30	2.65
35-54 years	3.42	1.61	2.61	1.69	1.86	2.36
55+ years	3.13	1.66	2.29	1.44	1.51	2.06
Anova (p-value)	2.03 (.133)	8.45 (<.001)	17.18 (<.001)	4.16 (.017)	17.08 (<.001)	4.91 (.008)
Education*						
School level - low	3.55	1.72	2.68	1.56	1.67	2.42
School level - high	3.32	1.92	2.78	1.65	2.00	2.32
University level	3.17	2.07	3.01	1.82	2.33	2.55
Anova (p-value)	2.61 (.076)	2.06 (.131)	1.60 (.204)	1.74 (.179)	8.72 (<.001)	0.63 (.532)
Ethnicity						
White	3.29	1.78	2.74	1.63	1.87	2.32
Other	3.57	2.44	3.06	1.96	2.45	2.87
t-test (p-value)	-1.79 (.075)	-3.40 (.001)	-1.81 (.071)	-2.34 (.020)	-3.79 (<.001)	-2.74 (.007)
Close friend or family member has had cancer						
No	3.53	1.93	3.12	1.85	2.14	2.66
Yes	3.28	1.90	2.70	1.64	1.93	2.34
t-test (p-value)	1.80 (.073)	0.18 (.858)	2.65 (.009)	1.64 (.103)	1.48 (.140)	1.69 (.093)
Social desirability						
Low score (0–5)	3.43	2.01	3.19	1.90	2.19	2.70
High score (6–10)	3.28	1.82	2.51	1.53	1.82	2.20
t-test (p-value)	1.17 (.242)	1.45 (.149)	5.11 (<.001)	3.21 (.002)	3.01 (.003)	3.12 (.002)

*Low school level = GCSEs as their highest qualification, High school level = A-levels or a qualification below degree.

## Discussion

This paper reports the development and validation of the Cancer Stigma Scale (CASS) designed to assess cancer stigma. The final scale includes 25 items assessing six different aspects of stigma; Awkwardness, Avoidance, Perceived severity, Policy Opposition, Personal Responsibility and Financial Discrimination. These components cover a range of aspects that are moderately correlated with one another, show adequate to good levels of internal and test-retest reliability, and fit well with the stigma literature. Mean scores were similar in the student and online-panel samples, supporting the validity of the scales.

The severity factor included items relating to how severe the consequences of a cancer diagnosis were expected to be and the likelihood of recovery from cancer. This fits well with Jones et al.’s [18] ‘course’ component and the ‘stability’ component identified by Weiner et al. [20]. Similarly, Sontag [45] identified the feeling of dread as a contributor to stigmatisation, stating that “treating cancer as no mere disease but a demonic enemy makes cancer not just a lethal disease but a shameful one” (p.59). The severity factor assesses the belief that a cancer diagnosis is catastrophic. Responses on the severity subscale may be amenable to change following raised awareness of the success of cancer treatments.

Personal Responsibility, which relates to how much a person’s actions are considered to have contributed to their cancer, has consistently been identified in stigma theory (sometimes referred to as ‘origin’ or ‘controllability’). Attributions of personal responsibility are made because the perceiver feels the need to explain why an event (in this case a diagnosis of cancer) has occurred [46], and some theories suggest that perceivers attribute responsibility to individuals to seek justification as to why they are experiencing illness (justification ideologies) [47]. Scores on the Personal Responsibility scale may increase as the public becomes more aware about lifestyle risk factors for cancer.

The cancer stigma scale also assessed Awkwardness, i.e. whether people feel comfortable around someone with cancer. Items tapping this aspect of stigma have been used in previous studies [45-47]. Anticipated awkwardness could be one reason why people would avoid interacting with someone that had cancer, and this is supported by the moderate correlation between Awkwardness and Avoidance (r = .43-.46). We found that people who reported more contact with someone who had had cancer had lower Awkwardness scores, suggesting some adaptation. Interventions designed to decrease the stigma of mental illness have shown that contact with people who have these illnesses is one of the most effective strategies [48]. Future work could consider the effect of similar interventions on perceived awkwardness around someone with cancer.

Avoidance, Policy Opposition and Financial Discrimination were also included in the scale. Goffman believed when a trait turns “those of us whom he meets away from him”, a person is the victim of stigmatisation in its purest form (p.15) [1], and this strongly supports the existence of the Avoidance component that is included in the scale. Avoidance items have also been included in many other studies that have assessed stigma of other illnesses e.g. [49-51].

The items on Financial Discrimination also fit well with studies in the context of other illnesses [29]. There are some legal boundaries in place to limit unfair discrimination against cancer patients (e.g. in the work-place), but in other instances discrimination is still acceptable (e.g. for obtaining travel insurance). While the estimation of a high chance of needing medical care is accurate in some cases, this is not always the case and making judgments on the basis of a cancer label alone, rather than an individual diagnosis, may not be considered fair. Financial worries can be an additional cause of stress following a cancer diagnosis, and fear of stigma can lead to patients avoiding claiming the financial benefits they are entitled to [52]. Understanding public opinion about such discrimination and how this correlates with other stigma components is an area for future research. The Policy Opposition component was correlated with Financial Discrimination. Although these sorts of items are less common in other stigma scales, they allowed us to include items tapping positive attitudes towards people with cancer.

Many of the stigma components were associated with socio-demographic factors. For the most part, being male was associated with higher cancer-related stigma (higher scores for Personal Responsibility, Avoidance, Policy Opposition and Financial Discrimination in at least one of the samples), as was younger age (higher scores for Personal Responsibility, Awkwardness, Avoidance, Policy Opposition and Financial Discrimination in at least one of the samples). In study 2, we also looked at ethnicity and education level. Being from an ethnic group other than white-British was associated with greater stigma (higher personal Responsibility, Avoidance, Policy Opposition, and Financial Discrimination) while being more educated was associated with higher Policy Opposition scores, but no other component.

Scoring higher on social desirability was associated with lower cancer-related stigma (lower Awkwardness, Avoidance, Policy Opposition and Financial Discrimination). This suggests that those who care more strongly about what others think of them are motivated to deny negative attitudes towards cancer patients. This association could suggest that participants may not be truthful when asked about cancer stigma and this has important implications for using the scale we have developed. We selected an internet-based survey to overcome the effects of social desirability, but it remained nevertheless and may have an even stronger influence if alternative collection methods were used.

There are other aspects of stigma theory that are not included in the CASS, for example we have not assessed the influence of concealability, disruptiveness or aesthetics; three components that are associated with the visible aspects of illness [19]. That is not to say they are irrelevant, but these other aspects may be specific to certain cancers/treatments (e.g. disfigurement following head and neck cancer), and in the interests of developing a brief, valid scale, we felt it best to focus on the most salient aspects of cancer stigma generally.

We acknowledge a number of limitations. As with all scales, the CASS is limited by the quality of the initial item pool. We attempted to ensure items had good face validity and covered all aspects identified by stigma theory by using items from previously validated measures and including several steps prior to factor analysis. Although our studies had adequate participants item ratios (1:7 for study 1 and 1:9 for study 2) in accordance with some criteria, others might recommend higher sample sizes. For convenience, we choose to use a student sample for the first part of this study. There are obvious limitations to the use of this sample, most notably their age and education level are not reflective of the general population. We did expand our work to include a general population sample following exploratory factor analysis, but neither of our studies was carried out in a representative sample, so the mean scores may not reflect those in the general population. In addition, using online administration means those who do not have access to or use computers were excluded. Regular internet use is associated with higher income [53] and our online panel sample (study 2) could reflect this.

Although there were a range of responses across the two samples, the results, particularly for some of the factors, were highly skewed. This is not surprising given the two samples were quite homogenous and we may expect more normally distributed responses if the data were collected in the general population. Severity scores did not vary by any of the variables measured which could suggest poor content validity or a very limited range of results. Future research should recruit samples that are expected a priori, to have high or low feelings of stigma. While this paper did consider experience of cancer, recruiting groups with particularly high levels of experience such as oncology nurses may generate more variation.

As is the case with development of any measure, our findings regarding the validity, reliability and structure of the CASS are limited to the context within which our data were collected. Additional work is needed to validate the CASS in different population sub-groups (e.g. ethnic minorities, lower literacy populations) and in different countries. Cancer is highly stigmatised in some cultures and the availability of a validated scale which could be used cross-culturally would be particularly useful.

Considering differences across cancers would also be interesting. The term cancer incorporates many diseases, and it is likely that some cancers are seen differently from others. We found that participants who rated lifestyle as the main contributing risk factor for cancer score more highly on the Personal Responsibility sub-scale. However, the conclusions we can draw from this are limited because the aetiology of each cancer is different. Previous work has shown that some cancers with an established behavioural aetiology attract greater blame attributions [22], and studies with patients and health professionals, suggest lung cancer attracts more stigma than other cancers as a result of its link with a behavioural cause (smoking) and poor prognosis [11]. A recent review suggested that anti-smoking campaigns, may contribute to stigma among lung cancer patients who smoke [11]. Changes in public perceptions of cancer stigma following such campaigns have not yet been assessed and the CASS could be a useful tool for doing this.

## Conclusions

This manuscript describes the development and validation of the CASS, a scale to assess cancer related stigma in a non-patient population. Stigma was first discussed in relation to cancer many years ago, but there has been little systematic research in this area. The availability of a tool to assess cancer stigma will help to identify if there is real cause for concern in this area and if so where interventions designed to decrease stigma are needed.

## Endnotes

^a^Cognitive interview procedures might be recommended at this stage, but given that our items were generated from previous work we felt it was acceptable to proceed without this.

^b^Because such a high proportion of participants in study 1 had indicated that a friend or family member had had cancer, these questions were changed slightly for study 2, so that they referred specifically to ‘close’ friends or relatives.

## Competing interests

The authors declare that they have no competing interests.

## Authors’ contributions

JW and LM conceived and designed the study. LM analyzed and interpreted the data. LM drafted the manuscript and JW critically revised the manuscript. Both authors have given final approval of the final version and agree to be accountable for all aspects of the work.

## Pre-publication history

The pre-publication history for this paper can be accessed here:

http://www.biomedcentral.com/1471-2407/14/285/prepub

## Supplementary Material

Additional file 1 Studies that informed the item pool. Click here for file
