# Supplementary material for: Development of a scale to assess cancer stigma in the non-patient population
Source: BMC Cancer. 2014 Apr 23;14:285. doi: 10.1186/1471-2407-14-285 (PMC4021096; doi:10.1186/1471-2407-14-285)
Supplement: Additional file 1 — Studies that informed the item pool. [file 1471-2407-14-285-S1.doc]

**Additional File 1: Studies that informed the item pool**

|  | **Source** | **Illness** | **Country** | **Items extracted** |
| --- | --- | --- | --- | --- |
|  | **Stigma measures review** [1] |  |  |  |
| [2] | Ramu, Dwivedi & Iyer (1975) | Leprosy | India | 7 |
| [3] | Myint et al (1992) | Leprosy | Myanmar | 8 |
| [4] | Raju & Kopparty (1995) | Leprosy | India | 8 |
| [5] | Van den Broek et al. (1998) | Leprosy | Tanzinia | 6 |
| [6] | Croft & Croft (1999) | Leprosy | Bangladesh | 4 |
| [7] | Shrum, Turner & Bruce (1989) | HIV/AIDS | USA | 54 |
| [8] | Mulford & Lee (1996) | HIV/AIDS | USA | 13 |
| [9] | O’Hea et al (2001) | HIV/AIDS | USA | 26 |
| [10] | Froman & Owens (2001) | HIV/AIDS | USA | 21 |
| [11] | Siyam’kela (2003) | Mental illness | South Africa | 14 |
| [12] | Taylor & Dear (1981) | Mental illness | Canada | 40 |
| [13] | Angermeyer & Matschinger (1996) | Mental illness | Germany | 25 |
| [14] | Corrigan et al. (2001a) | Mental illness | USA | 20 |
| [15] | Corrigan et al. (2001b) | Mental illness | USA | 6 |
| [16] | Weiner, Perry & Magnusson (1988) | Mental illness | USA | 13 |
| [17] | Angermeyer & Matschinger (2003) | Mental illness | Germany | 14 |
| [18] | Corrigan et al, (2004) | Mental illness | USA | 27 |
| [19] | Austin, Shafer & Deering (2002) | Epilepsy | USA | 7 |
| [20] | Neil (2001) | Skin disease | USA | 11 |
|  |  |  |  |  |
|  | **Stigma of cancer review** [21] |  |  |  |
| [22] | Chapple et al. (2004a) | Lung cancer | UK | 17 |
| [23] | Chapple et al. (2004b) | Lung cancer | UK | 2 |
| [24] | Tod et al. (2008) | Lung cancer | UK | 1 |
| [25] | Westerman et al. (2007) | Lung cancer | Netherlands | 2 |
| [26] | Peters-Golden (1982) | Breast Cancer | USA | 15 |
| [27] | Wilson & Lucker (2006) | Cancer | UK | 18 |
| [28] | Desnoo & Faithfull (2006) | Bowel Cancer | UK | 1 |
| [29] | Lam & Fielding (2003) | Breast Cancer | China | 4 |
| [30] | Macdonald & Anderson (1984) | Bowel Cancer | USA | 4 |
| [31] | Ohaeri et al (1998) | Breast/cervical Cancer | Nigeria | 5 |
| [32] | Sankar et al. (2006) | Breast Cancer | USA | 1 |
| [33] | Wong-kim et al. (2005) | Breast Cancer | USA | 3 |
| [34] | Ohaeri et al. (1999) | Breast/cervical Cancer | Nigeria | 2 |
| [35] | Ehrmann-Feldman et al. (1987) | Cancer | Canada | 1 |
| [36] | Miller et al. (2007) | Lung cancer | Kenya/USA | 15 |
| [37] | Schulte (2002) | Cancer | USA | 11 |
| [38] | Matthews et al. (2006) | Breast/cervical Cancer | USA | 1 |
| [39] | Azaiza & Cohen (2008) | Breast/cervical Cancer | Israel | 2 |
| [40] | Kwok, Sullivan & Cant (2006) | Breast Cancer | Australia | 2 |
| [41] | Kwok & Sullivan (2006) | Breast Cancer | Australia | 14 |
| [42] | Michielutte et al. (1996) | Skin cancer | USA | 1 |
|  |  |  |  |  |
| [43] | Berrenberg et al (1991) | Cancer | USA | 35 |
|  |  |  |  |  |
|  | **Total** |  |  | **481** |

**References**

[1] van Brakel WH: **Measuring health-related stigma--a literature review.** *Psychol Health Med* 2006, **11:**307-334.

[2] Ramu G, Dwivedi M, Iyer C: **Social reaction to leprosy in a rural population in Chingleput District (Tamil Nadu).** *Leprosy in India* 1975, **47:**156-169.

[3] Myint T, Thet A, Htoon M, Win M: **A comparative KAP study of leprosy patients and members of the community in Hlaing and Laung-Lon townships.** *Indian Journal of Leprosy* 1992, **64:**313-324.

[4] Raju M, Kopparty S: **Impact of knowledge of leprosy on the attitude towards leprosy patients: A community study.** *Indian Journal of Leprosy* 1995, **67:**259-272.

[5] van den Broek J, O'Donoghue J, Ishengoma A, Masao H, Mbega M: **Evaluation of a sustained 7-year health campaign on leprosy in Rufiji District, Tanzania.** *Leprosy Review* 1998, **59:**57-74.

[6] Croft R, Croft R: **Knowledge, attitude and practice regarding leprosy and tuberculosis in Bangladesh.** *Leprosy Review* 1999, **70:**34-42.

[7] Shrum J, Turner N, Bruce K: **Development of an instrument to measure attitudes toward acquired immune deficiency syndrome.** *AIDS Education and Prevention* 1989, **1:**222-230.

[8] Mulford C, Lee M: **Reliability and validity of AIDS victim blaming scales.** *Psychological Reports* 1996, **79:**191-201.

[9] O'Hea E, Sytsma S, Copeland A, Brantley P: **The attitudes toward women with HIV/AIDS scale (ATWAS): Development and validation.** *AIDS Education and Prevention* 2001, **13:**120-130.

[10] Froman R, Owens S: **Measuring attitudes toward persons with AIDS: The AAS-G as an alternate for of the AAS.** *Scholarly Inquiry for nursing practice* 2001, **15:**161-174.

[11] Siyam'kela Reference Groups. (2003). *HIV/AIDS stigma indicators. A tool for measuring the progress of HIV/AIDS stigma mitigation.* Cape Town: Policy Project, South Africa.

[12] Taylor SM, Dear MJ: **Scaling community attitudes toward the mentally ill.** *Schizophrenia Bulletin* 1981, **7:**225-240.

[13] Angermeyer M, Matschinger H: **The effect of personal experience with mental illness on the attitude towards individuals suffering from mental disorders.** *Social Psychiatry and Psychiatric Epidemiology* 1996, **31:**321-326.

[14] Corrigan PW, Green A, Lundin R, Kubiak MA, Penn DL: **Familiarity with and social distance from people who have serious mental illness.** *Psychiatric Services* 2001a, **52:**953-958.

[15] Corrigan PW, River LP, Lundin RK, Penn DL, Uphoff-Wasowski K, Campion J, Mathisen J, Gagnon C, Bergman M, Goldstein H, Kubiak MA: **Three strategies for changing attributions about severe mental illness.** *Schizophrenia Bulletin* 2001b, **27:**187-195.

[16] Weiner B, Perry RP, Magnusson J: **An attributional analysis of reactions to stigmas.** *Journal of personality and social psychology* 1988, **55:**738-748.

[17] Angermeyer M, Matschinger H: **The stigma of mental illness: effects of labelling on public attitudes towards people with mental disorder.** *Acta Psychiatrica Scandinavica 2003,* ***108:***304-309.

[18] Corrigan PW, Watson AC, Warpinski AC, Gracia G: **Stigmatizing attitudes about mental illness and allocation of resources to mental health services.** *Community Mental Health* 2004, **40:**297-307.

[19] Austin JK, Shafer PO, Deering JB: **Epilepsy familiarity, knowledge, and perceptions of stigma: report from a survey of adolescents in the general population.** *Epilepsy & Behavior* 2002, **3:**368-375.

[20] Neil JA: **The stigma scale: measuring body image and the skin.** *Plastic Surgical Nursing* 2001, **21:**79-82, 87.

[21] Lebel S, Devins GM: **Stigma in cancer patients whose behavior may have contributed to their disease.** *Future Oncol* 2008, **4:**717-733.

[22] Chapple A, Ziebland S, McPherson A: **Stigma, shame, and blame experienced by patients with lung cancer: qualitative study.** *British Medical Journal* 2004a, **328:**1470.

[23] Chapple A, Ziebland S, McPherson A, Summerton N: **Lung cancer patients' perceptions of access to financial benefits: a qualitative study.** *British Journal of General Practice* 2004b, **54:**589-594.

[24] Tod AM, Craven J, Allmark P: **Diagnostic delay in lung cancer: a qualitative study.** *Journal of Advanced Nursing* 2008, **61:**336-343.

[25] Westerman MJ, The AM, Sprangers MA, Groen HJ, van der WG, Hak T: **Small-cell lung cancer patients are just 'a little bit' tired: response shift and self-presentation in the measurement of fatigue.** *Quality of Life Research* 2007, **16:**853-861.

[26] Peters-Golden H: **Breast cancer: varied perceptions of social support in the illness experience.** *Social Science and Medicine* 1982, **16:**483-491.

[27] Wilson K, Luker KA: At home in hospital? **Interaction and stigma in people affected by cancer.** *Social Science and Medicine* 2006, **62:**1616-1627.

[28] Desnoo L, Faithfull S: **A qualitative study of anterior resection syndrome: the experiences of cancer survivors who have undergone resection surgery.** *European Journal of Cancer Care* 2006, **15:**244-251.

[29] Lam WW, Fielding R: **The evolving experience of illness for Chinese women with breast cancer: a qualitative study.** *Psychooncology* 2003, **12:**127-140.

[30] MacDonald LD, Anderson HR: **Stigma in patients with rectal cancer: a community study.** *Journal of Epidemiology & Community Health* 1984, **38:**284-290.

[31] Ohaeri JU, Campbell OB, Ilesanmil AO, Ohaeri BM: **Psychosocial concerns of Nigerian women with breast and cervical cancer.** *Psychooncology* 1998, **7:**494-501.

[32] Sankar P, Cho MK, Wolpe PR, Schairer C: What is in a cause? **Exploring the relationship between genetic cause and felt stigma.** *Journal of Medical Genetics* 2006, **8:**33-42.

[33] Wong-Kim E, Sun A, Merighi JR, Chow EA: **Understanding quality-of-life issues in Chinese women with breast cancer: a qualitative investigation.** *Cancer Control* 2005, **12:**6-12.

[34] Ohaeri JU, Campbell OB, Ilesanmi AO, Omigbodun AO: **The psychosocial burden of caring for some Nigerian women with breast cancer and cervical cancer.** *Social Science and Medicine* 1999, **49:**1541-1549.

[35] Ehrmann-Feldman D, Spitzer W, Del Greco L, Desmeules M: **Perceived discrimination against cured cancer patients in the workforce.** *Canadian Medical Association Journal* 1987, **136:**719-723.

[36] Miller AN, Fellows KL, Kizito MN. **The impact of onset controllability on stigmatization and supportive communication goals toward persons with HIV versus lung cancer: a comparison between Kenyan and U.S. participants.** *Journal of Health Communication* 2007, **22:**207-219.

[37] Schulte A: **Consensus versus disagreement in disease-related stigma: A comparison of reactions to AIDS and cancer patients.** *Sociological Perspectives* 2002, **45:**81-104.

[38] Matthews AK, Berrios N, Darnell JS, Calhoun E: **A qualitative evaluation of a faith-based breast and cervical cancer screening intervention for African American women.** *Health Education & Behavior* 2006, **33:**643-663.

[39] Azaiza F, Cohen M: **Between traditional and modern perceptions of breast and cervical cancer screenings: a qualitative study of Arab women in Israel.** *Psychooncology* 2008, **17:**34-41.

[40] Kwok C, Sullivan G, Cant R: **The role of culture in breast health practices among Chinese-Australian women.** *Patient Education and Counseling* 2006, **64:**268-276.

[41] Kwok C, Sullivan G: **Chinese-Australian women's beliefs about cancer: implications for health promotion.** *Cancer Nursing* 2006, **29:**E14-E21.

[42] Michielutte R, Dignan MB, Sharp PC, Boxley J, Wells HB: **Skin cancer prevention and early detection practices in a sample of rural women.** *Preventive Medicine* 1996, **25:**673-683.

[43] Berrenberg JL: **The Cancer Attitude Inventory: development and validation.** *Journal of Psychosocial Oncology* 1991, **9:**35-44.
